# Adipocyte-Derived *CCHamide-1*, *Eiger*, *Growth-Blocking Peptide 3*, and *Unpaired 2* Regulate *Drosophila melanogaster* Oogenesis

**DOI:** 10.3390/biom15040513

**Published:** 2025-04-01

**Authors:** Chad Simmons, Isaiah H. Williams, Tancia W. Bradshaw, Alissa Richmond Armstrong

**Affiliations:** Department of Biological Sciences, College of Arts and Sciences, University of South Carolina, Columbia, SC 29208, USA; simmonch@email.sc.edu (C.S.); isaiahhw@email.sc.edu (I.H.W.); tancia.bradshaw@nih.gov (T.W.B.)

**Keywords:** *Drosophila*, oogenesis, adipokines, inter-organ communication

## Abstract

In addition to energy storage, adipose tissue communication to other organs plays a key role in regulating organismal physiology. While the link between adipose tissue dysfunction and pathophysiology, including diabetes, chronic inflammation, and infertility, is clear, the molecular mechanisms that underlie these associations have not been fully described. We use *Drosophila melanogaster* as a model to better understand how adipose tissue communicates to the ovary. In this study, we utilized *D. melanogaster’s* robust genetic toolkit to examine the role of five adipokines known to control larval growth during development, *CCHamide-1*, *CCHamide-2*, *eiger*, *Growth-blocking peptide 3*, and *unpaired 2* in regulating oogenesis. We show that the adult fat body expresses these “larval” adipokines. Our data indicate that ovarian germline stem cell maintenance does not require these adipokines. However, adipocyte-derived *CCHamide-1*, *eiger*, *Growth-blocking peptide 3*, and *unpaired 2* influence early and late germline survival as well as ovulation. Thus, this work uncovers several adipokines that mediate fat-to-ovary communication.

## 1. Introduction

Obesity, recognized as a disease in the US by the American Heart Association in 2013, is a growing concern for many countries in the world [1]. In 2018, one-third of all adults in the US were considered obese [2], and the rates are projected to increase to around 50% by 2030 [3]. Adults and children with obesity are at a higher risk of developing type 2 diabetes, cardiovascular disease, and several cancers [4]. With obesity, the imbalance between energy uptake (overeating) and expenditure (reduced activity) causes increased lipid storage in adipose tissues and lipid accumulation in non-adipose tissues [5]. Obesity-associated mitochondrial dysfunction, inflammation, and oxidative stress in the adipose tissue disrupt metabolism and the function of other organs [4,6,7,8,9]. Perturbations at the cellular level negatively impact organ and tissue function, leading to comorbidities such as infertility [10], cardiovascular dysfunction [11], and the development of non-alcoholic fatty liver disease [12]. While adipose tissue is a major mediator of inter-organ communication, the cellular and molecular mechanisms that underlie this association are not well understood. We use the model organism *Drosophila melanogaster* to uncover how adipose tissue communicates to other organs.

*Drosophila melanogaster* is a robust model system that is widely used to study inter-organ communication [13]. Many biological pathways are conserved between humans and flies, with around 85% of human disease-causing genes having homologs in *Drosophila* [14,15]. Additionally, the genetic toolkit of *Drosophila* allows for tissue- and cell type-specific manipulation of gene expression [16]. The *Drosophila* fat body, composed primarily of adipocytes, has similar energy storage and endocrine roles as mammalian adipose tissue [17]. In addition to being used as a model for diet- and genetic-associated obesity [18,19], several *Drosophila* studies have begun to elucidate the mechanisms employed by the fat body to communicate to other tissues [20]. Not surprisingly, *Drosophila* adipose tissue serves as a central nutrient-sensing depot that relays information about dietary input to the highly nutrient-responsive ovary.

The *Drosophila* fat body employs nutrient-sensing pathways, nuclear hormone receptors, nutrient transport proteins, and metabolic enzymes to remotely control the diet-dependent process of oogenesis [21,22,23,24,25,26,27,28]. The *Drosophila* ovary is made up of 16 to 20 ovarioles, with each one being an individual egg-producing unit containing the progressive stages of oocyte development [29]. The apical most part of the ovariole, the germarium, contains germline stem cells (GSCs) whose undifferentiated state is maintained by adherence to and signaling from cap cells (CCs) [30]. GSCs divide asymmetrically to self-renew and generate differentiated cystoblasts that synchronously divide four times with incomplete cytokinesis to form a 16-cell germline cyst. A single-cell layer of epithelial follicle cells surrounds each 16-cell cyst that buds from the germarium to form an individual egg chamber. One of the cells in the cyst becomes the oocyte, while the remaining 15 cells become nurse cells that support oocyte development. Each ovariole contains six to eight developing egg chambers [30], with the most developed egg chamber, or mature oocyte, at the posterior end being ready for ovulation and fertilization. Previous studies showed that GSC maintenance, germline cyst survival, progression through vitellogenesis, and ovulation are regulated by nutrient-sensing pathways, including mechanistic Target of Rapamycin (mTOR), the amino acid response pathway (AAR), insulin/insulin-like growth factor (IIS), and Ras/MAPK signaling [21,23,24]. However, the fat body-derived factors that mediate the adipose tissue control of oogenesis are unknown.

In humans, adipose tissue secretes adipokines, such as leptin, tumor necrosis factors, and interleukins to carry out endocrine control of multiple aspects of physiology, such as controlling nutritional intake and insulin sensitivity and moderating inflammatory responses [4,31,32]. During development in *Drosophila*, the fat body secretes a variety of adipokines that control overall growth and developmental timing, similar to human adipocyte signaling [20,33,34]. Nutrient-sensitive neuropeptides, CCHamide-1 (*CCHa1*) and CCHamide-2 (*CCHa2*), are produced in the gut and fat body [35]. *CCHa1* interacts with the anterior dorsal neuron and the pigment-dispersing factors in the brain to regulate sleep cycles [36] and stabilize circadian behavioral rhythms [37]. *CCHa2* binds to its receptor in the brain (*CCHa2*R) to promote *Drosophila* insulin-like peptide (dILP) production [38], with mutations in *CCHa2* and *CCHa2*R resulting in larval growth defects [38]. *Drosophila* tumor necrosis factor, eiger (*egr*), is released from the fat body in response to starvation [39]. During starvation, *egr* binds to its receptor Grindelwald (grnd), expressed in the brain, to suppress *dILP* expression [39] and to Wengen (wgn), expressed in gut enterocytes, to restrict lipid catabolism and maintain tissue homeostasis [40]. Growth-blocking peptides (*Gbp*) regulate immune responses [41] and stimulate *dILP* production by acting on insulin-producing cells (IPCs) in the brain. The knockdown of *Gbp*s in the larval fat body led to smaller-sized adults [41]. Unpaired 2 (*upd2*) is secreted from the fat body in response to dietary fats and sugars [42]. Similarly to human leptin, *Drosophila upd2* is upregulated with elevated fat stores and is downregulated during reduced nutritional conditions [43,44]. *Upd2* regulates how much insulin is released into the circulation from IPCs [45]. Given their role in controlling growth during larval development, we proposed that these adipokines may also mediate the fat body control of oogenesis during adulthood. Here, we show that the adult fat body expresses *CCHa1*, *CCHa2*, *egr*, *Gbp3*, and *upd2*. Adipocyte-specific knockdown of these adipokines leads to a general increase in triglyceride storage. We found that none of these adipokines are required for proper GSC maintenance. However, our evidence indicates that *egr*, *upd2*, and *CCHa1* are important for germline survival, while *Gbp3* promotes ovulation. This work identifies fat-derived factors that modulate specific steps of oogenesis, thus filling in the knowledge gap of how adipose tissue relays information to other organs.

## 2. Materials and Methods

### 2.1. Drosophila Strains and Culture Conditions

Fly lines used for this study were obtained from the Bloomington *Drosophila* Stock Center (BDSC). The following transgenic fly lines were used for these series of experiments: RFP-RNAi (31417), *CCHa1*-RNAi (#57562), *CCHa2*-RNAi (#57183), *egr*-RNAi (#55276 and #58993), *upd2*-RNAi (#33949 and #33988), and *Gbp3*-RNAi (#64108). We used the driver line *tubPGal80ts;Lsp2(3.1)Gal4/TM6B* for temperature-sensitive adipocyte-specific knockdown [21] and the *w^1118^* line to stimulate oogenesis with our selected progeny. Canton S and Oregon R fly lines were used to determine adipokine presence in adult fat. Expanded stocks were kept at room temperature (20–25 °C) on a molasses medium (Archon Scientific, Durham, NC, USA) and flipped weekly. Molasses medium consisted of 86% water, 0.574% agar, 6.3% cornmeal, 1.52% yeast, 4.65% dry molasses, 0.39% propionic acid, 0.15% methylparaben, and 0.52% ethanol (Archon Scientific).

### 2.2. Adipocyte Specific Manipulation of Gene Expression

Crosses were set up in triplicate, with each bottle containing 20 to 30 virgin adult female *tubPGal80^ts^;3.1Lsp2-Gal4/TM6B* flies and 10 adult male UAS-transgene-RNAi flies. UAS RFP-RNAi was used as a control in each cross. Flies were kept on a molasses medium diet supplemented with wet yeast paste. Wet yeast paste was composed of 2 parts inactive dry yeast to 1 part deionized water. Crosses were kept at 18 °C and flipped every four days until progeny emerged. At least 20 virgin female flies of the appropriate genotype (*tubPGal80^ts^;Lsp2(3.1)Gal4 > UAS-target-RNAi*) were collected per adipokine knockdown target over three days. To stimulate oogenesis, *w^1118^* adult males were added to target female progeny. These experimental flies were maintained at 18 °C for four days to allow for clearance of larval fat before moving to 29 °C for 10 days to induce adipokine knockdown in adipocytes. Therefore, ovaries were harvested from female flies who were between 14 and 17 days old at the time of dissection.

### 2.3. Ovary Immunostaining and Fluorescence Microscopy

After 10 days of transgene expression, ovaries from adult target female flies were dissected in PBS. Ovaries were fixed in 5.3% paraformaldehyde (PFA) (Electron Microscopy Sciences, Hatfield, PA, USA) in PBS for 13 min. The ovaries were rinsed twice in 0.5% Triton-X (VWR Life Sciences, Radnor, PA, USA) in PBS (PBT) and washed three times on a nutator for 15 min. Samples were placed in a blocking solution (5% Bovine Serum Albumin, 5% Normal Goat Serum, and 0.5% Trition-X in PBS) and incubated for at least 24 h. After blocking, primary antibodies mouse alpha spectrin (2 µg/mL, Developmental Studies Hybridoma Bank (DHSB), Iowa City, IA, USA), mouse anti-Lamin C (2 µg/mL DSHB), and rabbit anti-cleaved Dcp-1 (1:250, Cell Signaling Technology, Danvers, MA, USA) were diluted in blocking buffer and added to the samples. After overnight incubation, the primary antibody solution was removed, and the samples were washed three times for 10 min each in PBT. A secondary antibody solution containing Alexa-Fluor anti-mouse 488, Alexa-Fluor anti-rabbit 568, and blocking solution were added to each sample (1:250 each), and the samples were incubated for two hours shielded from light. Once the secondary antibody solution was removed, the samples were washed 3 times in PBT for 15 min each and protected from the light. After the last wash, all the PBT was removed, and the samples were stored in Vectashield with 4′,6-diamidino-2-phenylindole (DAPI). Ovaries were mounted onto glass slides with glass coverslips before analyzing on a confocal microscope.

### 2.4. Adipocyte Immunostaining and Fluorescence Microscopy

Abdominal carcasses were collected from adult female target progeny after 10 days of transgene activation. The gut, ovaries, and malpighian tubes were removed from the carcasses, leaving only the fat body attached to the carcass. Insect dissection pins (Austerlitz) were used to anchor the corners of the carcasses to the bottom of a Sylgard-coated (DOW Chemical, Midland, MI, USA) twelve-well tissue culture dish. The carcasses were fixed for 20 min in 5.3% PFA in PBS. At room temperature, samples were rinsed twice and washed three times for 15 min in 0.1% Tween-20 in PBS (PBT). Abdominal carcasses were incubated overnight in blocking solution (5% NGS, 5% BSA, 0.1% PBT in PBS). The carcasses were then incubated with 3 µg/mL mouse anti-alpha spectrin (DSHB) diluted in blocking solution overnight. Samples were then washed three times for 15 min in 0.1% PBT before a 2 h incubation period while being protected from light with anti-mouse Alexa-Fluor 568 diluted in blocking solution (1:250). To visualize lipid droplets, samples were washed three times in 0.1% PBT before a 30 min incubation period with BODIPY 505/510 (25 ng/mL) at room temperature while being protected from light. Samples were then washed with 0.1% PBT, and abdominal carcasses were stored in Vectashield containing DAPI. Fat body tissues were removed from the abdominal carcass onto slides prior to imaging.

### 2.5. Ovarian Analysis

All ovarian analysis was performed using a Zeiss LSM 800 confocal microscope (Carl Zeiss Microscopy, LLC, Thornwood, NY, USA) using ZEN 2.6 software. Using 63× magnification, cap cells (CC) were counted using nuclear morphology and Lamin C staining. Germline stem cells (GSC) were counted using alpha spectrin labeling of the fusome. Dcp-1+ germaria were counted based on the presence of Dcp-1 in any of the cysts present in the germaria. GSC, CC, and Dcp-1+ germaria were counted at 0 and 10 days of transgene expression. At least 3 biological replicates were analyzed with 50 to 150 germaria counted per timepoint, per sample in each replicate. Rates of GSC loss were statistically verified using two-way ANOVA with interaction (GraphPad Prism 8). Shifts in rates of Dcp-1+ germaria from the control were analyzed using Student’s t-test for statistical significance between the control and transgene samples. A 20× magnification was used to analyze the presence of dying vitellogenic follicles in the ovary samples. The total number of complete ovarioles was counted, and the total number of ovarioles that contained Dcp-1 positive staining or pyknotic nuclei at or after stage 8 of vitellogenesis was recorded. The percentages of dying vitellogenic follicles (DVFs) in each transgene group were tabulated and analyzed using Student’s t-test against the control group.

### 2.6. Blocked Ovulation Analysis

After 4 days of incubation in 18 °C, 15 to 20 adipokine knockdown progenies were placed into vials with male *w^1118^* flies in molasses media supplemented with wet yeast. Flies were then placed in a 29 °C incubator and flipped daily. After the tenth day, the ovaries were dissected from the female progenies and analyzed for blocked ovulation. An ovary was considered blocked if it had more than one stage 14 egg chamber present in any of the ovarioles. A percentage was determined using the number of blocked ovaries against the total number of ovaries counted. At the time of dissection, the flies were between 14 and 17 days old. Student’s *t*-test was used to determine whether there were statistically significant changes between the transgene samples and the control.

### 2.7. Measurement of Adipocyte and Lipid Droplet Size

Using a confocal microscope, z-stack images were taken from fat body samples to capture multiple images at a 1 µm depth for adipocyte size and lipid droplet size analysis. Using ZEN Blue lite 2.8 software and ImageJ (ImageJ2 version number: 2.14.0/1.54f), measurements were taken using a stylus for the largest areas present in the fat body according to alpha spectrin staining. Around 10–15 measurements were taken per fat body for a total of around 50–150 measurements per sample analyzed. The measurements were averaged, and an ordinary one-way ANOVA was performed to compare the control group to each adipokine knockdown group. ImageJ was also used to determine lipid droplet size for each of the fat bodies analyzed. The lipid droplets were made visible by BODIPY staining. Each lipid droplet per sample was measured by automated segmentation using the threshold selection method in ImageJ. The background of each image was subtracted, and the threshold was determined based on a setting that resembled the edges of the lipid droplets. The total measurements were averaged in each biological replicate, and an ordinary one-way ANOVA was performed to compare the percentages of each bin for each knockdown to the corresponding bin of the control.

### 2.8. Adipocyte Bradford and Triglyceride Assays

After 10 days of transgene expression, the abdominal carcasses of control and transgene female progeny were dissected. Carcasses were placed in a 1.5 mL microcentrifuge tube and submerged in a triglyceride lysis buffer (140 mM NaCl, 50 mM Tris-HCl, and 0.1% Triton-X in water with protease inhibitor cocktail [Research Products International, Mt Prospect, IL, USA] added). A pestle was used to grind carcasses in lysis solution. Samples were then centrifuged, and the supernatant was removed and placed into a clean, labeled microcentrifuge tube. The Bradford assay and triglyceride assay were performed using the Bradford Method Protein Assay kit (M173-KIT) (VWR, Radnor, PA, USA) and Stanbio Liquicolor Triglycerides Kit (STANBIO 2100-225) (VWR, Radnor, PA, USA). Measurements were performed in triplicate using SoftMaxPro 6.4 software on a SpectraMax i3 Plate Reader (Molecualr Devices, San Jose, CA, USA). For each biological replicate, all values were converted to µg/mL. Triglyceride-to-protein ratios were determined by dividing the amount of triglycerides by the amount of protein. Fold change was calculated by dividing the triglyceride/protein ratio of each transgenic sample to the ratio of its RFP-RNAi control for that biological replicate. Student’s *t*-test was used to determine whether the fold changes from the transgenic groups were statistically significant compared to the control groups.

### 2.9. RNA Isolation, RT-PCR, and qPCR

After 10 days of transgene expression at 29 °C, the ovary, gut, and malpighian tubes were removed from the abdomen in PBS to obtain isolated abdominal carcasses. Carcasses with fat bodies attached were stored in RNA Shield (Zymo Research, Irvine, CA, USA). RNA was isolated from each sample using the Quick RNAi Mini-Prep kit (Zymo Research). An amount of 100 ng of RNA was used to synthesize cDNA using the Verso cDNA synthesis kit (ThermoFisher Scientific, Waltham, MA, USA). RNA and cDNA quantities and A260/A280 quality were measured using Smartdrop L spectrophotometer (Accuris Instruments, Edison, NJ, USA). Primers were derived from previously published RT-PCR and qPCR work and FlyPrimerBank (Table S1). Canton S, IV, and Oregon R adult fat body cDNA samples were used for RT-PCR quantification of adipokine presence in adult fat. RT-PCR quantification was performed using Econotaq PLUS GREEN (Lucigen, Middleton, WI, USA) on an iCycler Thermocycler (Bio-Rad, Hercules, CA, USA). Alpha tubulin primers were used as a control. Knockdown efficiency was quantified using SYBR Green reagents (PowerTrack SYBR Green Master Mix—Applied Biosciences) and a Quantstudio 3 Real-Time PCR System (Applied Biosystems, Thermofisher Scientific). Relative quantification of at least three biological replicates was performed using the comparative Ct method. Student’s *t*-test was used to determine the statistical significance between the knockdown samples and the control samples.

## 3. Results

### 3.1. Adipokines with Roles in Larval Development Are Expressed in the Adult Fat Body

**Figure 1:**
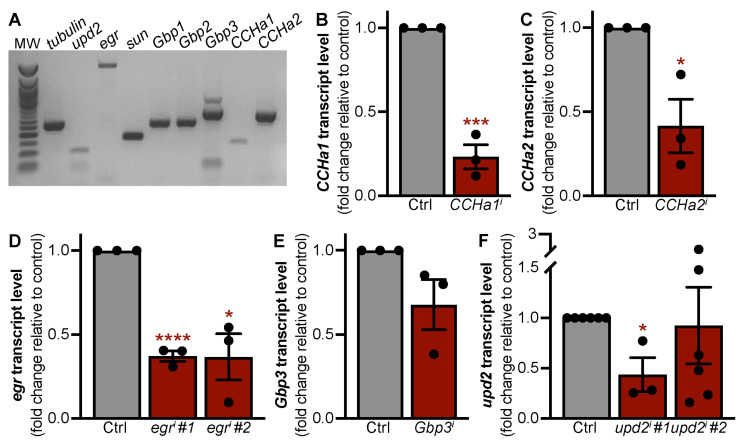
RNAi-mediated knockdown of adipokines expressed in adult adipocytes. (**A**) RT-PCR analysis of larval adipokine expression in adult female fat bodies (MW = molecular weight ladder; tubulin used as loading control). (**B**–**F**) qRT-PCR analysis of mRNA expression in fat body tissue from females with RNAi-mediated knockdown of *CCHa1* (**B**), *CCHa2* (**C**), *egr* (**D**), *Gbp3* (**E**), and *upd2* (**F**) compared to RFP-RNAi control (Ctrl). Data shown as mean ± SEM. * *p* < 0.05, *** *p* < 0.001, **** *p* < 0.0001, no indicator = not significant. Student’s two-tailed *t*-test was used. Original images can be found in Figure S1.

We first asked whether the fat bodies of adult females express adipokines known to have a role in larval development. Using RT-PCR, we detected transcripts for *unpaired 2* (*upd2*); *eiger* (*egr*); *stunted* (*sun*); *growth-blocking peptides 1*, *2,* and *3* (*Gbp1*, *Gbp2*, and *Gbp3*); *CCHamide-1* (*CCHa1*); and *CCHamide-2* (*CCHa2*) (Figure 1A). To determine whether these adipokines regulate oogenesis, we used the adipocyte-specific *tubP-Gal80^ts^;3.1Lsp2-Gal4* driver (*3.1Lsp2^ts^*) [21] to express *UAS-dsRNA transgenes* targeting each adipokine for RNAi-mediated knockdown. Given that nutrient-sensing pathways like IIS and mTOR have been previously shown to function within the fat body to modulate oogenesis [21,23], we focused our analysis on *CCHa1*, *CCHa2*, *egr*, *Gbp3*, and *upd2* based on their known roles in relaying nutrient status [35,38,39,40,41,42]. To determine knockdown efficiency, we used qRT-PCR to measure the transcript levels of each adipokine in adult female fat bodies from control and adipokine knockdown flies (Figure 1B–F). Compared to the controls, there was significant knockdown for all UAS-dsRNA transgenic lines: *CCHa1* (76.7%) (Figure 1B), *CCHa2* (58.4%) (Figure 1C), *egr* line 1 (*egr#1*—62.8%), *egr* line 2 (*egr#2*—63.2%) (Figure 1D), and *upd2* line 1 (*upd2 #1*—56.3%) (Figure 1F). Two RNAi lines showed a moderate to slight reduction in transcript levels: *Gbp3* (32.2%) (Figure 1E) and *upd2* line 2 (*upd2#2*—7.7%) (Figure 1F). Of the six trials assessing the *upd2* transcript level using the second *UAS-dsRNA transgene*, there were two outliers showing overexpression, thus dampening the effect of knockdown (Tables S2 and S3). Upon outlier exclusion, the upd2 transcript level using the second UAS-dsRNA transgene was reduced by 62.4%.

### 3.2. CCHa1, CCHa2, Egr, Gbp3, and Upd2 Do Not Cell-Autonomously Control Adipocyte or Lipid Droplet Size

**Figure 2:**
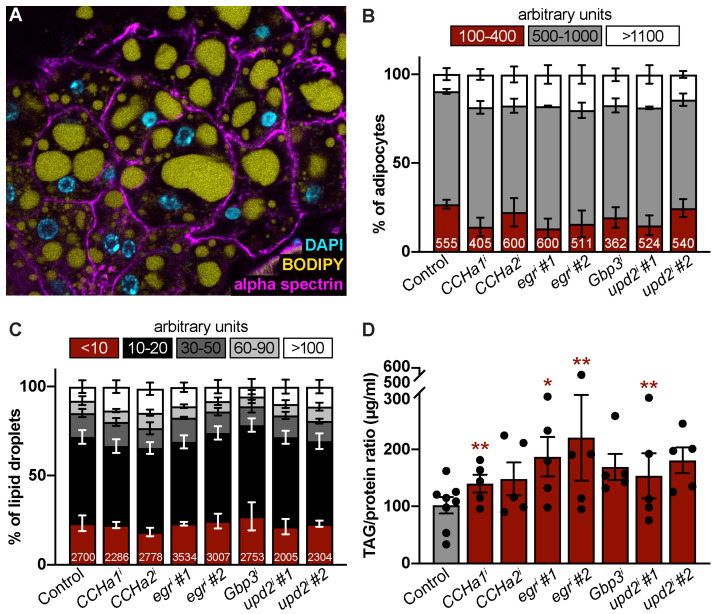
*CCHa1*, *CCHa2*, *egr*, *Gbp3*, and *upd2* control lipid storage but not adipocyte size. (**A**) Representative image of adipocytes (40×) from females labeled with alpha spectrin (magenta, cell membranes), BODIPY (yellow, lipid droplets), and DAPI (cyan, nuclei). (**B**) Percentage of adipocytes within small (100–400 bin), medium (500–1000), and large (greater than 1100) size bins measured in arbitrary units. Total number of adipocytes measured per sample indicated at bottom of each bar. Error bars represent SEM across biological replicates for each bin. No statistical significance observed between controls and target knockdown sample bins as assessed via ordinary one-way ANOVA with multiple comparisons (each knockdown condition compared to control). (**C**) Percentage of lipid droplets within very small (less than 10), small (10–20), medium (30–50), large (60–90), and very large (greater than 100) size bins measured in arbitrary units. Error bars represent SEM across biological replicates for each bin. No statistical significance observed between controls and target knockdown sample bins as assessed via ordinary one-way ANOVA with multiple comparisons (each knockdown condition compared to control). (**D**) Fold change triglyceride-to-protein ratio relative to control for each adipokine knockdown from five biological replicates (individual data points). Data are presented as mean ± SEM. * *p* < 0.05, ** *p* < 0.01, no indicator = not significant. Student’s two-tailed *t*-test was used.

Based on previous work, changes in adipocyte size can be observed by feeding *Drosophila* a high-fat diet [46] and by knocking down certain proteins in the fat body [47]. We asked whether our subset of adipokine targets controls adipocyte cellular biology by using whole-mount adult fat body immunocytochemistry [48] to assess cell and lipid droplet size (Figure 2A). In the control and adipokine knockdown fat bodies, most adipocytes fall within the mid-range size (Figure 2B). While the average percentage of large adipocytes tends to be higher with *CCHa1*^RNAi^ (18.5% ± 3.1), *CCHa2* ^RNAi^ (17.7 ± 4.5), *egr* #1 ^RNAi#1^ (18% ± 5.3), *egr*#2^RNAi#1^ (20.2% ± 5.3), *Gbp3* ^RNAi^ (17.3% ± 3.6), *upd2* ^RNAi#1^ (18.8% ± 5.2), and *upd2* ^RNAi#2^ (14.2% ± 1.9) compared to the control (9.7% ± 3.4), there is no significant difference in average adipocyte size (Figure 2B and Table S4). Similarly, lipid droplet sizes, ranging from very small to very large, were comparable across the control and *CCHa1*, *CCHa2*, *egr*, *Gbp3*, and *upd2* knockdown fat bodies (Figure 2C and Table S5). We also measured levels of triglycerides, the major form of stored fat in *Drosophila* adipocytes [49]. The adipocyte-specific knockdown of each adipokine resulted in higher TAG levels compared to the control, with *CCHa1*, *egr*, and *upd2* knockdown showing statistically significant increases (1.7- to 2-fold change) (Figure 2D and Table S6). Taken together, these data suggest that the *CCHa1*, *egr*, and *upd2* adipokines regulate lipid storage in adult female adipocytes.

### 3.3. Adipocyte-Derived CCHa1, CCHa2, Egr, Gbp3, and Upd2 Do Not Regulate Ovarian GSC Maintenance

**Figure 3:**
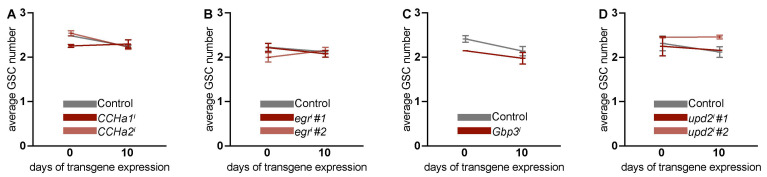
Adult adipocyte-specific knockdown of *CCHa1*, *CCHa2*, *egr*, *Gbp3*, and *upd2* does not affect ovarian GSC number. Average number of GSCs was counted in ovaries from control group and from adipocyte-specific RNAi groups *CCHa1^i^* and *CCHa2^i^* (**A**), *egr^i^* (**B**), *Gbp3^i^* (**C**), and *upd2^i^* (**D**). Data are presented as mean ± SEM for two to seven biological replicates. Total number of germaria counted for 0 and 10 days of transgene expression is given in Table S7. No statistically significant differences in GSC number were observed using two-way ANOVA with interaction.

The genetic knockdown of amino acid receptors [21] and scavenger receptors [28] led to a reduction in the number of GSC in the germarium. Removing adipocyte-specific factors from the IIS and TOR signaling pathway has had detrimental effects on GSC maintenance and the process of vitellogenesis [21,23,24]. We asked whether *CCHa1*, *CCHa2*, *egr*, *Gbp3*, and *upd2* in adult adipocytes control the ovarian GSC number. As observed in previous studies [21,23,24], the average GSC number declines slightly with age (Figure 3). Upon the RNAi-mediated knockdown of *CCHa1*, *CCHa2*, *egr*, *Gbp3*, and *upd2* in adipocytes, the GSC loss associated with age was comparable to that of controls (Figure 3A–D and Table S7). Interestingly, we observed that the GSC number remained stable, i.e., it did not decrease with age, with *CCHa1* and *upd2* knockdown in adipocytes; however, there was no statistically significant difference compared to the controls (Figure 3A,D). Therefore, we conclude that *CCHa1*, *CCHa2*, *egr*, *Gbp3*, and *upd2* originating from adipocytes do not support GSC maintenance.

### 3.4. Adipocyte-Derived CCHa1, CCHa2, Egr, Gbp3, and Upd2 Do Not Support Early Germline Survival or Progression Through Vitellogenesis

**Figure 4:**
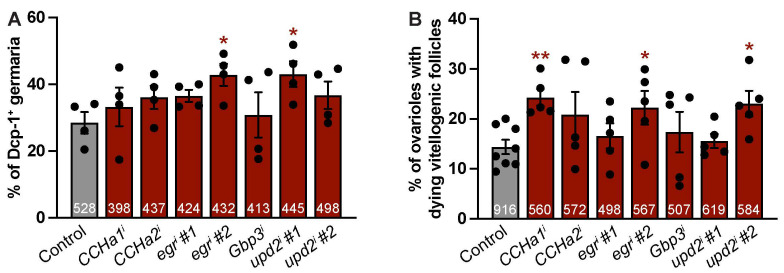
Adipocyte-derived *CCHa1*, *egr*, and *upd2* promote germline survival at nutritional checkpoints. (**A**) Percentage of germaria with Dcp-1 immunoreactivity in germline after 10 days of adipokine knockdown. Total number of germaria analyzed over four independent trials indicated at bottom of each bar. Data are shown as mean ± SEM. * *p* < 0.05 according to Student’s two-tailed *t*-test. (**B**) Percentage of ovarioles with dying vitellogenic follicles based on Dcp-1 positive immunoreactivity after 10 days of adipokine knockdown. Number of ovarioles examined over five independent trials are shown at bottom of each bar. Data are shown as mean ± SEM. * *p* < 0.05, ** *p* < 0.01, no indicator = not significant. Student’s two-tailed *t*-test was used.

Dietary input impacts germline survival early during oogenesis, at the 16-cell cyst stage in the germarium, as well as later during the progression of egg chambers through vitellogenesis [50]. Previous studies have shown that this is in part mediated by communication from the adipose tissue [22,23,24,25,26,27]. For example, the adipocyte-specific knockdown of components of the IIS pathway, from the receptor to key players in the downstream effector axes, leads to reduced GSC maintenance [23,24]. Therefore, we asked whether *CCHa1*, *CCHa2*, *egr*, *Gbp3*, or *upd2* function within adipocytes to support the survival of the early germline. We quantified germline death in germaria from flies in which individual adipokines had been knocked down in adult adipocytes. On average, the percentage of Dcp-1^+^ germaria was slightly higher with the adipocyte knockdown of *CCHa1*, *CCHa2*, *egr*, and *upd2*, but not *Gbp3*, when compared to the control (Figure 4A and Table S8), with statistically significant increases observed for *egr* and *upd2*. Of note, while both RNAi lines for *egr* and *upd2* showed an increased percentage of Dcp-1^+^ germaria, only one line for each reached statistical significance. Next, we asked whether these adipokines function within adipocytes to support the survival of the germline later during oogenesis by measuring the number of vitellogenic egg chambers undergoing cell death. On average, the percentage of ovarioles containing a dying vitellogenic egg chamber was higher than that of the control for the adipocyte knockdown of *CCHa1*, *egr*, and *upd2*, but not for *CCHa2* or *Gbp3* (Figure 4B). Unexpectedly, the *upd2*-#1 RNAi line did not show an effect on vitellogenesis, while the *upd2*-#2 RNAi line had a statistically significant effect on the increase in dying vitellogenic follicles. For the adipokines that showed higher levels of vitellogenic egg chamber cell death, an approximate 10% increase was observed (Table S9). Altogether, these data indicate that adipocyte-derived *egr* and *upd2* support the survival of early and late germlines, while adipocyte-derived *CCHa1* predominantly promotes vitellogenesis.

### 3.5. Adipocyte-Derived Gpb3 Promotes Ovulation of Mature Oocytes

**Figure 5:**
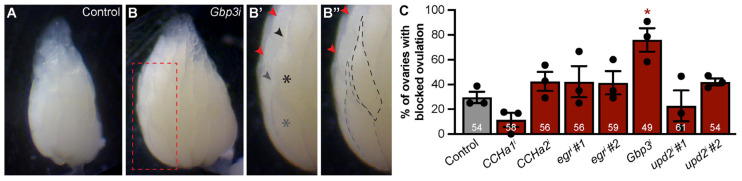
*Gbp3* in adipocytes promotes ovulation. (**A**–**B″**) Representative stereomicroscope images taken at 3× of ovaries from controls (**A**) and *Gbp3* knockdown (**B**) females. (**B′**) Region highlighted by dashed outline in B magnified, labeling two oocytes (gray and black asterisks) in one ovariole and their corresponding dorsal appendages (gray and black arrowheads, respectively). Dorsal appendages for another ovariole containing two mature oocytes are marked with red arrowheads. (**B″**) Same image as (**B′**) but with two foreground oocytes in one ovariole indicated by dashed outlines. (**C**) Percentage of ovaries showing blocked ovulation. Total number of ovarioles counted over three biological replicates indicated inside each bar. Data are shown as mean ± SEM. * *p* < 0.05, no indicator = not significant. Student’s two-tailed *t*-test was used.

Individual ovarioles in adult *Drosophila* female ovaries contain only one or zero mature oocytes because of continual ovulation (Figure 5A), the last step of oogenesis that is sensitive to nutritional input [50]. Moreover, ovulation is regulated by mTOR signaling, the integrated stress response, and Ras/MAPK activity within adult adipocytes [21,24,25]. When single ovarioles retain more than one mature oocyte, ovulation is considered to be blocked [21] (Figure 5B,B′). To determine whether *CCHa1*, *CCHa2*, *egr*, *Gbp3*, or *upd2* could mediate fat body control of ovulation, we quantified ovulation block in samples with the adipocyte-specific knockdown of each adipokine. For the knockdown of *CCHa1*, *CCHa2*, *egr*, and *upd*2, the percentage of ovaries with mature oocyte retention was comparable to that of the controls (Figure 5C). However, the adipocyte-specific knockdown of *Gbp3* resulted in approximately a 45% increase in blocked ovulation compared to the controls (Figure 5A–C and Table S10). Thus, adipocyte-derived *Gbp3* is required to promote ovulation.

## 4. Discussion

A substantial body of work provides evidence that the fat body relays nutritional information to the ovary [21,22,23,24,25,26,27]. While the fat body secretes many factors [51], the identity of fat body-derived factors that communicate to the ovary are unknown. We took clues from studies of larval development that have identified several adipokines shown to regulate overall growth and postulated that *CCHa1*, *CCHa2*, *egr*, *Gbp3*, and *upd2* might mediate fat-to-ovary communication observed in adult females. This work continues to advance the understanding of how the fat body remotely controls oogenesis by uncovering adipokines that regulate several aspects of oogenesis.

### 4.1. The Fat Body Utilizes Multiple Adipokines to Regulate the Ovarian Germline Stem Cell Lineage

We observed from an earlier study that the ovary is very sensitive to nutritional changes. Depending on the diet fed to flies, the amount of egg production varies. High-sugar diets cause *Drosophila* to lay less eggs over time and increase the number of dying vitellogenic follicles [52]. The changes in diet are facilitated by nutrient signaling pathways, such as insulin and insulin-like growth factor signaling, amino acid sensing, and TOR signaling [53]. The knockdown of components of these pathways, such as the insulin receptor (InR) [23] and Ras/MAPK pathway components [24], led to significant increases in cell death in the germaria. *egr*, *upd2*, and *CCHa2* are adipokines that communicate with their own respective receptors in the brain to control the amount of dILP secreted into the hemolymph [38,39,41,43]. Based on our data, the adipokines examined in this study are not required for ovarian germline stem cell maintenance. However, our work indicates that individual adipokines have specific roles in controlling stem cell lineage. Additional known and unknown nutrient-responsive adipokines may compensate for each other. Therefore, a limitation of this study is the single adipokine knockdown approach. A combinatorial knockdown approach would provide more information on the potential compensatory nature. Moreover, another system for the manipulation of gene expression, such as CrispR/Cas9 [54], may need to be used to completely remove a specific adipokine from the fat body.

### 4.2. Direct Communication from Adipocytes to the Ovary

Adipocyte-to-ovary communication might be mediated by adipokines acting directly on ovarian cells. In order to interact with the ovary in a direct manner, we would expect ovarian cells to express adipokine receptors as follows: CCHa1-R and CCHa2-R for CCHamides [38,55], wgn and grnd for eiger [56], methuselah-like 10 (mthl10) for Gbp3 [57,58], and domeless (dome) for upd2 [59]. According to the Fly Cell Atlas, ovarian somatic and/or germline cells express all of these receptors to some degree [60]. Interestingly, mthl-10 is robustly expressed and seemingly enriched in follicle cells. Mthl10 is a G-protein-couple receptor that mediates its functions using the PLC/Ca2+ intracellular signaling pathway [57]. Given that calcium signaling contributes to the process of ovulation [61], adipocyte-derived Gbp3 might control ovulation (Figure 5) by binding to mthl10 expressed in follicle cells and promoting calcium signaling. Dome also shows strong expression, particularly in follicle cells and ovarian germline cysts [62,63]. Dome is a cytokine receptor that mediates its functions using the JAK/STAT intracellular signaling pathway [59]. JAK/STAT activity has a well-known role in border cell migration during *Drosophila* oogenesis [62]. DIAP1, a JAK/STAT pathway target, controls cell survival in the *Drosophila* testis [64]. Therefore, adipocyte-derived upd2 might control the survival of early and late germlines (Figure 4) by binding to dome expressed in the ovary.

### 4.3. Indirect Communication from Adipocytes to the Ovary

Alternatively, adipocyte-to-ovary communication might be indirect with adipokines acting on other tissues that then relay information to the ovary. Insulin-like peptides (ILPs) secreted from insulin-producing cells (IPCs) in the brain are known to control germline stem cells and their progeny [65]. Interestingly, CCHa2-R, but not CCHa1-R, is expressed in larval but not adult IPCs [35]; however, dopaminergic neurons in the adult brain express CCHa2-R [66]. Similarly, larval IPCs express grnd, one of the receptors for egr [39]. Both CCHa2-R knockout and grnd knockdown lead to the retention, i.e., perturbed secretion, of ILPs [38,39]. Given that insulin/insulin-like growth factor signaling functions in the ovary to control germline cyst survival and progression through vitellogenesis [67], adipocyte-derived CCHamides and egr might control the survival of early and late germlines (Figure 4) by controlling ILP secretion from brain IPCs. Future studies will delineate how these adipokines communicate to the ovary.

### 4.4. The Partial Knockdown of Target Adipokines from the Fat Body May Not Be Enough to Influence Ovarian Homeostasis

The knockdown of nutrient signaling adipokines in fat led to an increase in triglyceride levels but did not cause any significant changes to the morphology of the adipocyte. On average, all adipokine target knockdowns led to increases in the triglyceride/protein ratio when compared to the control (Figure 2D). For some targets like *upd2*, this is unusual, as *upd2* is secreted by the fat body in response to dietary fat and sugar. *upd2* activates JAK/STAT signaling in GABAergic neurons to stop the inhibition of IPCs, which then secrete dILPs to promote growth and fat storage [42,43,44]. *upd2* reduction in the fat would slow IPC dILP production, which should result in smaller flies with less triglyceride levels [42]. As noted, we find that knocking down *upd2* with one RNAi line shows an effect on germline cyst death but not on the death of vitellogenic follicles, while the reverse is true for the second *upd2* RNAi line (Figure 4). Perhaps this discrepancy can be explained by off-target effects, residual upd2, and the differential sensitivity of early (germline cysts) versus late (vitellogenic follicles) germline survival.

Nutrient sensing is vital for maintaining growth and energy storage in both humans and in *Drosophila*. Understanding how adipocytes signal nutrient status to other tissues can improve clinical research and spur developments and treatments for obesity and its associated comorbidities. Through this work, we examined how reductions in key signaling adipokines in the fat can alter the maintenance and physiology of the ovary. The adipocyte-specific knockdown of our target adipokines did not lead to drastic amounts of cell death or disruption but statistically influenced aspects of germline stem cell homeostasis, vitellogenesis, triglyceride storage, and ovulation. Future studies should aim to determine whether there are compensatory mechanisms in nutrient signaling. We also observed that *Gbp3* adipocyte-specific knockdown may destabilize egg-laying processes and block ovulation. Investigating the effect of *Gbp1* and *Gbp2* adipocyte-specific knockdown on the ovary may provide more insights as to how those adipokines modulate ovarian processes outside of nutrient status.

## 5. Conclusions

Using the *Gal80^ts^/Gal4/UAS* gene expression system to knock down each adipokine in adult adipocytes, we found that *CCHa1*, *egr*, *Gbp3*, and *upd2* have roles in controlling specific nutrient-sensitive stages of oogenesis. We show that GSC maintenance is not regulated by any of the adipokines tested (Figure 3). However, *egr* and *upd2* regulate the survival of early germline cysts, and along with *CCHa1*, they regulate the survival of vitellogenic egg chambers (Figure 4). Lastly, we found that *Gbp3* specifically controls ovulation (Figure 5). *CCHa2* did not play a role in any of the stages of oogenesis examined.

## Data Availability

The original contributions presented in this study are included in the Supplementary Excel workbook. Further inquiries can be directed to the corresponding author.

## Supplementary Materials

The following supporting information can be downloaded at: https://www.mdpi.com/article/10.3390/biom15040513/s1. Figure S1: Original image for Figure 1A. The following supporting tables can be found in the form of sheets in an Excel workbook: Table S1: qPCR and RT-PCR primer lists; Table S2: qPCR relative quantification values; Table S3: Expanded qPCR data for comparative Ct experiments; Table S4: Average adipocyte size binned distributions and ANOVA; Table S5: Lipid droplet bin distributions and ANOVA; Table S6: Triglyceride and Bradford fold changes; Table S7: GSC and CC count summarization; Table S8: Average Dcp-1-positive germaria counts; Table S9: Average number of dying vitellogenic follicles; Table S10: Blocked ovulation averages.
