# Supplementary material for: Adipocyte-Derived CCHamide-1, Eiger, Growth-Blocking Peptide 3, and Unpaired 2 Regulate Drosophila melanogaster Oogenesis
Source: Biomolecules. 2025 Apr 1;15(4):513. doi: 10.3390/biom15040513 (PMC12024527; doi:10.3390/biom15040513)
Supplement: Supplementary file 1 [file biomolecules-15-00513-s001.zip › Simmons Biomolecules Figure S1.pdf]

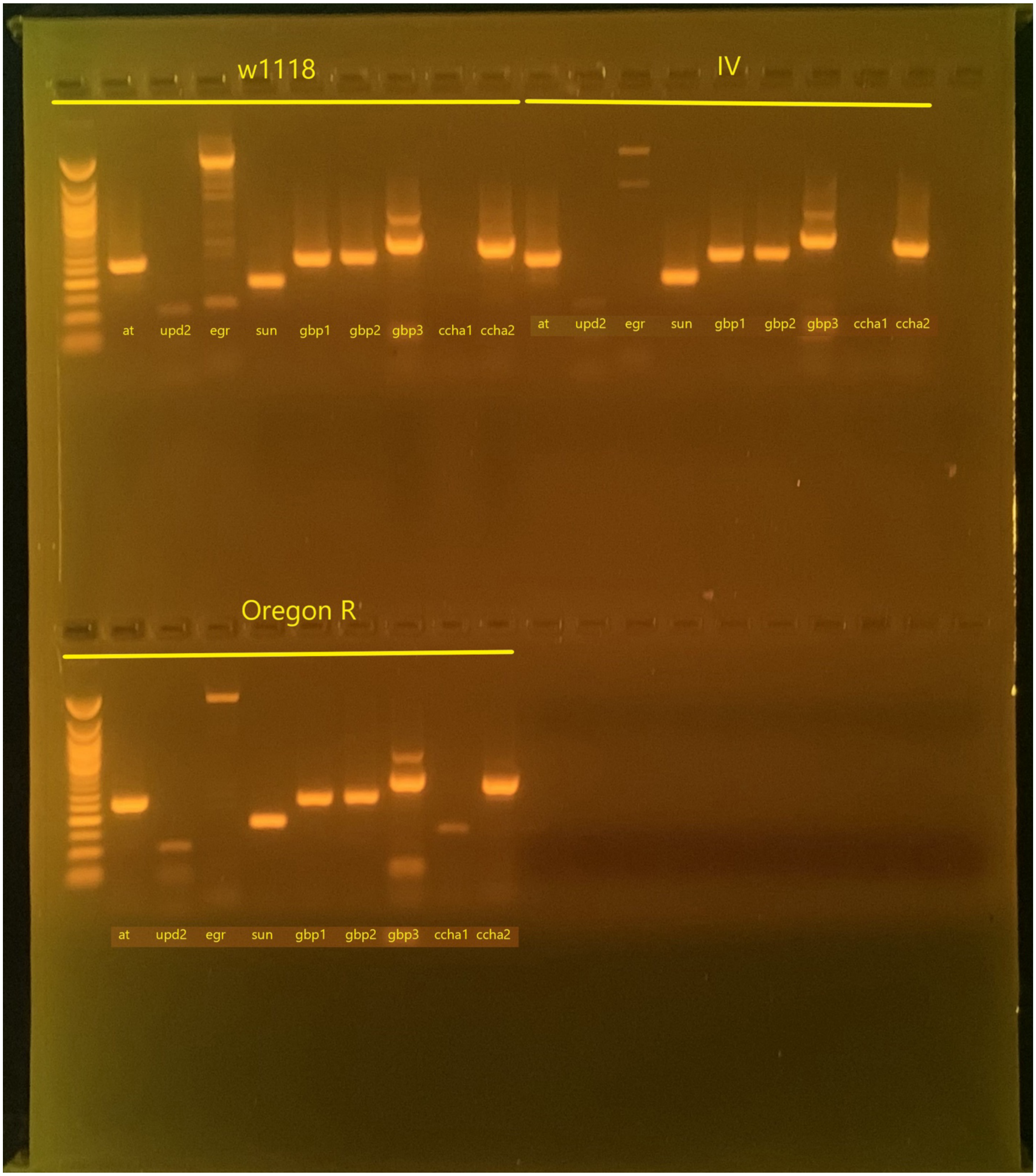

Figure S1. Original image for Figure 1A. RT-PCR analysis of larval adipokine expression in adult female fat bodies from the following backgrounds - w^{1118}, IV, and Oregon R (at, alpha-tubulin used as loading control).
